# Building Extraction Based on an Optimized Stacked Sparse Autoencoder of Structure and Training Samples Using LIDAR DSM and Optical Images

**DOI:** 10.3390/s17091957

**Published:** 2017-08-24

**Authors:** Yiming Yan, Zhichao Tan, Nan Su, Chunhui Zhao

**Affiliations:** Department of Information Engineering, Harbin Engineering University, Harbin 150001, China; zhaochunhui@hrbeu.edu.cn

**Keywords:** stacked sparse autoencoder, LIDAR DSM, remote sensing image, building extraction

## Abstract

In this paper, a building extraction method is proposed based on a stacked sparse autoencoder with an optimized structure and training samples. Building extraction plays an important role in urban construction and planning. However, some negative effects will reduce the accuracy of extraction, such as exceeding resolution, bad correction and terrain influence. Data collected by multiple sensors, as light detection and ranging (LIDAR), optical sensor etc., are used to improve the extraction. Using digital surface model (DSM) obtained from LIDAR data and optical images, traditional method can improve the extraction effect to a certain extent, but there are some defects in feature extraction. Since stacked sparse autoencoder (SSAE) neural network can learn the essential characteristics of the data in depth, SSAE was employed to extract buildings from the combined DSM data and optical image. A better setting strategy of SSAE network structure is given, and an idea of setting the number and proportion of training samples for better training of SSAE was presented. The optical data and DSM were combined as input of the optimized SSAE, and after training by an optimized samples, the appropriate network structure can extract buildings with great accuracy and has good robustness.

## 1. Introduction

Nowadays, aerial optical images and digital surface model (DSM) obtained from Light detection and ranging (LIDAR) are main high resolution data for citizien remote sensing applications [1,2,3,4].

As the most typical features of artificial landscapes, buildings play an important role in urban planning, urban development and military affairs [5].

How to extract buildings quickly and accurately from high-resolution remote sensing data has become the primary problem that needs to be studied. With the rapid development of remote sensing technology, multiple sensors remote sensing data also show high-resolution features. This provides a good condition for the analysis of interested objects [6]. However, complex information in the images brings negative effects to building extraction. The traditional remote sensing image building extraction methods can be summarized as the following three categories [7]: (1) The line and corner extraction-based methods. These methods usually first draw straight lines on the basis of the straight line, and then the lines are grouped, merged and removed, and finally screens out the exact outline of the building [8,9,10]; (2) Region segmentation—based methods-these methods extract the image characteristics, and then get the building areas. For example, the texture feature of the building can be obtained by using the gray level co-occurrence matrix, the gray-difference matrix and the Gabor filter of the extracted image, and the extraction of the building is realized by the segmentation of the texture feature [11]; (3) Methods based on auxiliary features or information [12,13,14]. Due to the apparent height difference between the building areas and the ground, the building areas are judged by the height between the buildings and other surrounding objects. These traditional building extraction methods typically utilize a single optical image or DSM data, and these methods have their own limitations. The methods based on geometric boundaries have the problem of not making full use of texture features and spatial features [15], which leads to extraction errors, and these methods construct polyhedral building models to judge the building or to fit the building, but this model for the appearance of continuous or complex building adaptability is not strong, and the robustness is poor [16].

The region segmentation-based methods depend on the accuracy of segmentation, and the error is difficult to avoid which is caused by the stacking and shadowing of the buildings. Also there are some classification based methods as image fitting methods, random forests supervised hierarchical classification were also used and obtained an good results [17,18,19]. Building extracts based on DSM auxiliary data are highly expensive for data acquisition [2,18,19]. The large-scale acquisition cost of such data is too high and the update cycle is uncertain.

In addition to the above method, after the gradual development, some methods from the building model have been proposed. These methods are based on multi-dimensional high-resolution remote sensing data, starting from the semantic model of buildings. The model-driven building extraction methods mainly includes the method based on semantic model classification with a prior knowledge model.

With the introduction of the Bayesian network and probability graph model, some researchers also use probabilistic latent semantic analysis (PLSA) [20] and latent Dirichlet allocation (LDA) theme models [21] to carry out the work. On the other hand, the Markov Random Field (MRF) [22], Conditional Random Field (CRF) [23], and multi-scale random field, etc., are based on image primitives (pixels or split regions). The spatial relationship between the building models was used for high-resolution remote sensing image building object extraction. In addition, many researchers have attempted to use the curve evolution technique, including snakes or active contour models, snakes deformation model, and level set, based on a priori model for building extraction.

Based on the classification of the semantic model, the spatial relationship between objects and the background is still insufficient. The precision of the extraction needs to be improved. Due to the variety of artificial buildings, the dependence on the prior knowledge is large when the building model is established. It is difficult to find a pervasive model to describe; based on the “wide range of priority” of the visual cognitive theory of the method is currently only stay in the simple object recognition and extraction.

In order to improve the effectiveness of building extraction, it is possible to extend and integrate the existing methods or models of training by means of incremental learning. It is worth noting that the intelligent algorithm of machine learning can be used to optimize and adapt the model parameters. With the rapid development of theoretical methods of machine learning, such as the emergence of manifold learning and sparse expression, the depth of learning theory and other methods in the field of image processing and artificial intelligence has been widely used.

As a result, properly combining an optical image with DSM data can be a better choice. The sparse autoencoder network has a very good feature learning ability. We present a Stacked Sparse Autoencoder (SSAE) framework [24] to extract the buildings in the high-resolution remote sensing images. However, training sample selection and network structure settings of the SSAE have a significant impact on its accuracy. For better building extraction using optical images and DSM together, our contributions are these: (1) a better setting strategy of SSAE network structure is given; (2) an idea of setting the number and proportion of training samples for better training of SSAE is presented. The optical data and DSM were combined as an input of the optimized SSAE, and after training by an optimized samples, the appropriate network structure can extract buildings with great accuracy and has good robustness. The following of the paper is set as: Section 2 reports the theory and method of the optimized SSAE. In Section 3, experimental analysis and results are shown. In Section 4, the work is concluded and discussed.

## 2. Methods

### 2.1. The Framework

**Figure 1:**
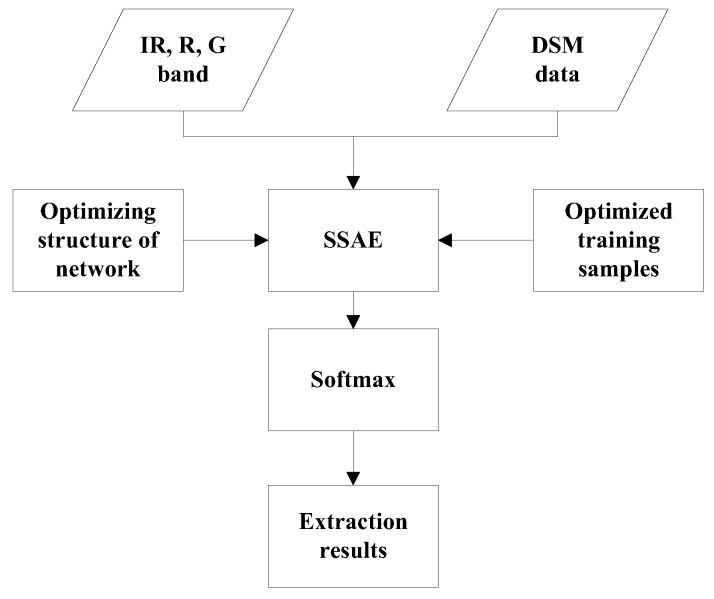
The framework of the building extraction method.

The framework is shown in Figure 1. First, the three bands of optical data and DSM data were input into a stacked sparse autoencoder, and then the training samples were analyzed and studied to determine the appropriate size and proportion of training samples. After obtaining the appropriate training samples, analysis the influences of the number of hidden neurons and the number of hidden layers on the accuracy. Then the appropriate training samples and structure of SSAE is obtained which can be used for better building extraction.

The greedy layer wise approach was employed for pre-training stacked sparse autoencoder by training each layer in turn [25]. After the pre-training, the trained stacked sparse autoencoder will be employed to building and non-building patches extraction in testing set. All layers were combined together to form a stacked sparse autoencoder with hidden layers and a final softmax to extract buildings pixels from high-resolution remote sensing images and DSM.

### 2.2. Stacked Sparse Autoencoder

The stacked sparse autoencoder is an unsupervised feature learning algorithm. Stacked sparse autoencoder has all the advantages of the depth network and more powerful expression, tends to learn the characteristic representation of the input data. For a stacked sparse autoencoder, the first layer can learn the first-order features, the second can learn second-order features, etc.

**Figure 2:**
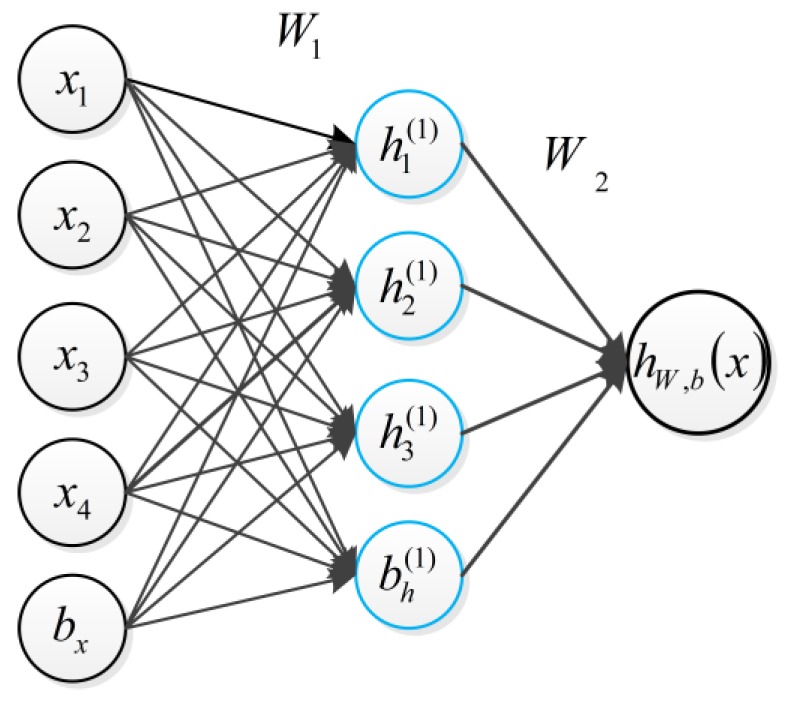
A simple example of an autoencode.

Basically, training an autoencoder is to find the optimal parameters by minimizing the discrepancy between input *x* and its reconstruction *x*ˆ. As shown in Figure 2. The encoder is the input *x* to the implicit representation of the mapping of h, expressed as

(1) $$h\;=\;f\left(x\right)\;=\;S_{f}\left(Wx\;+\;b\right)$$

Here *S*() is activation function which are defined by the sigmoid logistic function:

(2) $$\begin{array}{l} h_{1}^{\left(1\right)}\;=\;S_{f}\left(W_{11}^{\left(1\right)}x_{1}\;+\;W_{12}^{\left(1\right)}x_{2}\;+\;W_{13}^{\left(1\right)}x_{3}+\;b_{1}^{\left(1\right)}\right)\\ h_{2}^{\left(1\right)}\;=\;S_{f}\left(W_{21}^{\left(1\right)}x_{1}\;+\;W_{22}^{\left(1\right)}x_{2}\;+\;W_{23}^{\left(1\right)}x_{3}\;+\;b_{2}^{\left(1\right)}\right)\\ \cdots \end{array}$$

The final expression is represented by $W_{ji}^{\left(l\right)}$, and offset $b^{\left(l\right)}$, *L* is the *L*th layer, *j* is the *j*th node:

(3) $$h_{W,b}\left(x\right)\;=\;S_{f}\left(W_{11}^{\left(2\right)}h_{1}^{\left(2\right)}\;+\;W_{12}^{\left(2\right)}h_{2}^{\left(2\right)}\;+\;W_{13}^{\left(2\right)}h_{3}^{\left(2\right)}\;+\;\cdots +\;b_{1}^{\left(2\right)}\right)$$

The discrepancy between input *x* and its reconstruction *x*ˆ is described with a cost function:

(4) $$J\left(W,b\right)\;=\;\left[\frac{1}{m}\sum_{i\;=\;1}^{m}J\left(W,b;x\left(i\right),y\left(i\right)\right)\right]\;+\;\frac{\lambda }{2}\sum_{l\;=\;1}^{n_{i}-1}\sum_{i\;=\;1}^{s_{i}}\sum_{j\;=\;1}^{s_{i}+1}{\left(W_{ji}^{l}\right)}^{2}$$

(5) $$=\;\left[\frac{1}{m}\sum_{i\;=\;1}^{m}\left(\frac{1}{2}{\left\|h_{w,b}\left(x\left(i\right)\right)\;-\;y\left(i\right)\right\|}^{2}\right)\right]\;+\;\frac{\lambda }{2}\sum_{l\;=\;1}^{n_{i}\;-\;1}\sum_{i\;=\;1}^{s_{i}}{\sum_{j\;=\;1}^{s_{i}\;+\;1}\left(W_{ji}^{\left(l\right)}\right)}^{2}$$

The first term in the above formula is the mean square error term, and the second term is the regularization term (also called the weight attenuation term).

We use the bulk gradient descent method to solve the objective function. Each iteration in the gradient descent method is required to update the parameters. The reverse derivative algorithm is used to calculate the partial derivative, after obtaining the loss function and its partial derivative, the gradient reduction algorithm is used to solve the optimal parameters of the network.

The stacked sparse autoencoder neural network is a neural network composed of multi-layer sparse self-encoders, the outputs of each layer is wired to the inputs of the successive layer, and the same method to train all the hidden layer, with the output of the last hidden layer as a multi-classifier soft-max input with the original data tag to train the soft-max classifier network parameters. Finally these network parameters as the entire neural network parameters, including all the hidden layers and a soft-max output layer, then find the minimum value for the price function parameters, and this is the entire network of the optimal parameter values.

**Figure 3:**
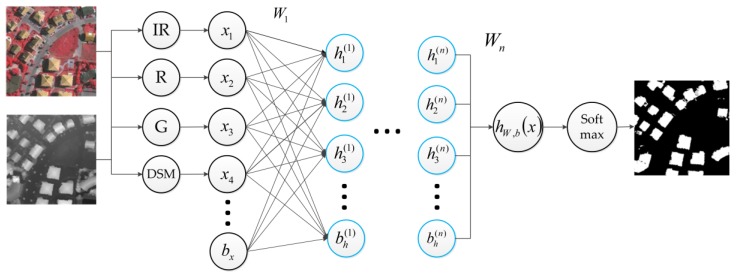
The illustration of the stacked sparse autoencoder used in this work.

As shown in Figure 3, we use such a stacked sparse autoencoder, with optical image and DSM data as the input, to extract the buildings.

### 2.3. Evaluation

The extraction results of different methods were evaluated as ‘*Accuracy*’. ‘*Accuracy*’ is a measure of the correct point to all points:

(6) $$Accuracy\;=\;\frac{T}{N}$$

*T* is the point that is correctly extracted, including the number of building pixels that had been correctly extracted and the number of non-building pixels that had been correctly extracted. *N* represents all the points in this image.

The extraction results of different methods were also evaluated in terms of ‘*Completeness*’ ‘*Correctness*’ and ‘*quality*’, defined as in Equations (7)–(9):

(7) $$Completeness\;=\;\frac{TP}{TP+FN}$$

(8) $$Correctness\;=\;\frac{TP}{TP+FP}$$

(9) $$Quality\;=\;\frac{1}{\frac{1}{Completeness}\;+\;\frac{1}{Correctness}\;-\;1}$$

In these equations, *TP*, *FN*, and *FP* are the numbers of true position, false negative, and false positive, respectively.

Here the true position is the number of building pixels that had been correctly extracted based on the ground truth. False negative, and false positive are defined in similar ways.

## 3. Experiments and Analysis

### 3.1. The Generation of Training and Testing Sets

In this experiment, several groups of optical images and DSM were collected as the input data to a stacked sparse autoencoder. Digital aerial images are used as experimental data. This digital aerial images are a part of the high-resolution DMC block of the German Association of Photogrammetry and Remote Sensing (DGPF) test. Including the near infrared (IR) spectral band, red (R) spectral band and green (G) spectral band, the spatial resolution is 9 cm. At the same time provide matching DSM data and real orthographic projection (standard true map), which DSM data spatial resolution of 9 cm. The ground truth map has a spatial resolution of 9 cm and a data quantization of eight bits.

We selected digital aerial images and their DSM data in Area 30 and Area 37 as the training data set. The luminance of buildings in Area 30 is in the low range, while the luminance of the buildings in Area 37 color is high. The size of each image is 1500 pixels × 1500 pixels. In the first layer or input layer, the input is the raw pixel intensity of square patch which is represented as column vectors of pixel intensity its size is 2,250,000 pixels × 4 floors. The four bands include the IR, the R, the G, and the DSM, and all are normalized.

**Figure 4:**
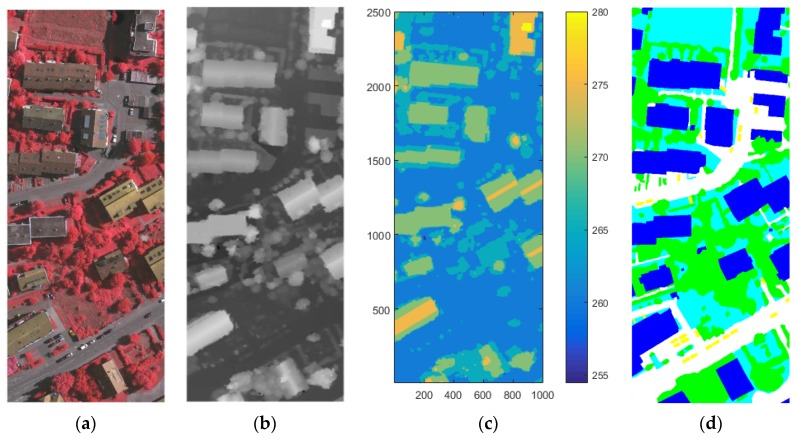
The Vaihingen test area: (**a**) Area 34; (**b**) the ALS DSM data of Area 34; (**c**) the contour of the Area 34; and (**d**) the ground truth map of Area 34.

**Figure 5:**
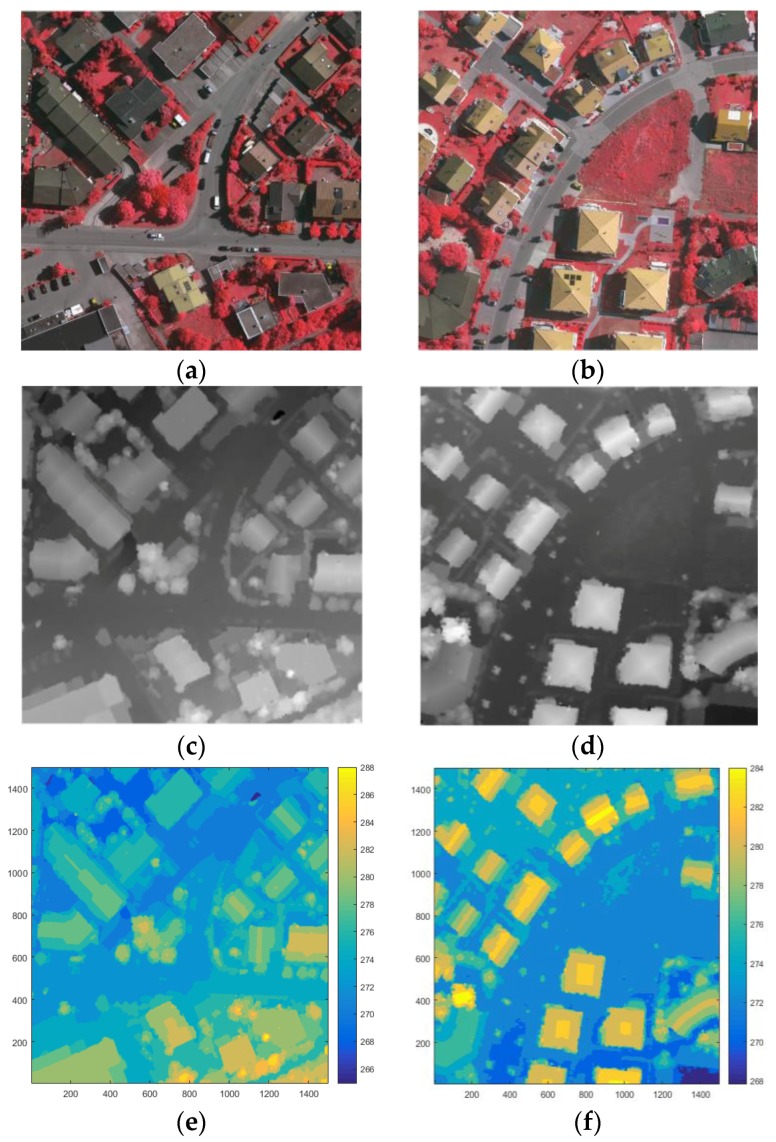

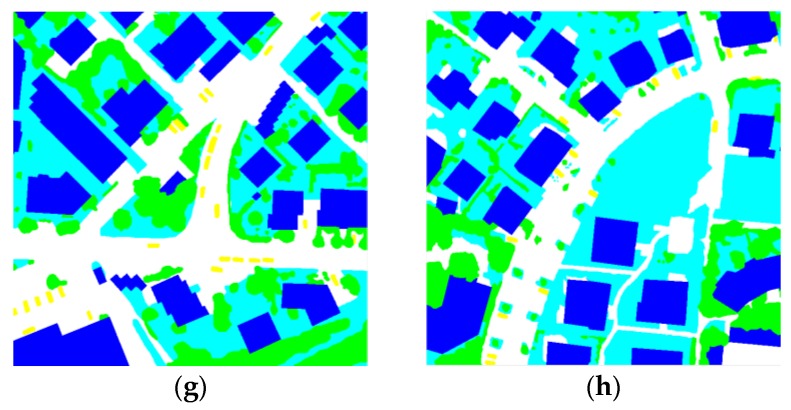
The Vaihingen test area: (**a**) Area 30; (**b**) Area 37; (**c**) the DSM of Area 30; (**d**) the DSM of Area 37; (**e**) the contour of the Area 30; (**f**) the contour of the Area 37; (**g**) the ground truth map of Area 30; and (**h**) the ground truth map of Area 37.

Area 34 was selected as test data, since the buildings in Area 34 are of various luminance. The digital aerial images and DSM were also used. The size of this images is 2500 pixels × 1000 pixels, and is represented as column vectors of pixel intensity whose size is 2,500,000 pixels × 4 floors. Likewise, the four layers include the IR band, the L band, the G band, and the DSM, and all are normalized. Moreover, it can be found in the contours of each areas that the terrains have ups and downs, as shown in Figure 4c and Figure 5e,f, and some ground in the high-altitude areas have larger values of DSM than the roof of the buildings that in low-altitude areas. This can be used to verify the performance of our methods in different cases of terrain.

### 3.2. Impact of the Different Proportion of Training Samples

A total of 100,000 training samples were randomly selected from the 30th and 37th regions, and the neural networks were trained according to different proportions. Each image has 225,000 pixels, and the 30th and 37th regions have a total of 4,500,000 pixels. The selected building pixels to non-building pixels ratio was different for the next experiment. Using a network structure with two hidden layers. The first and second hidden layers have 200 and 100 hidden units, respectively.

**Figure 6:**
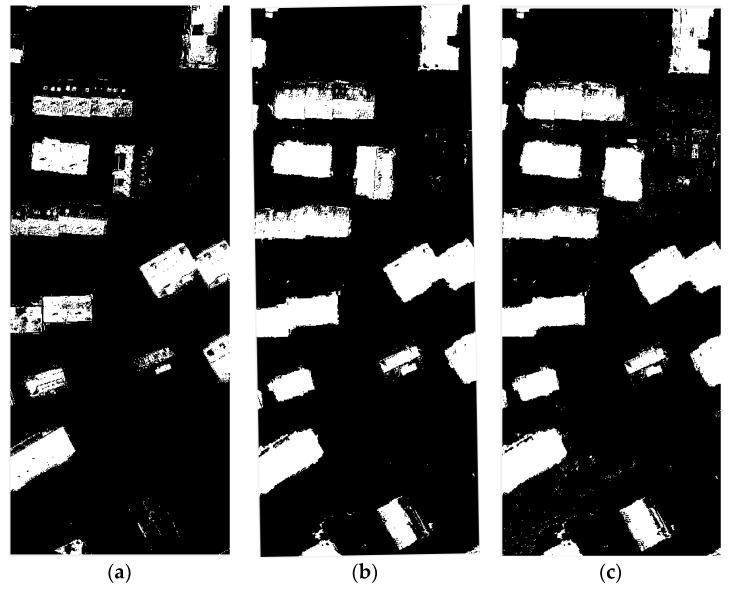

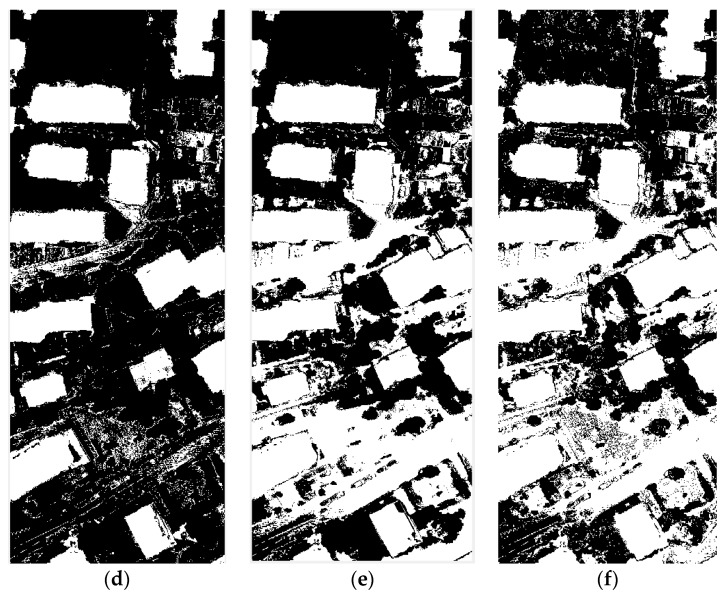
Different proportions of extraction results: (**a**) the ratio of buildings to non-buildings is 2 to 8; (**b**) the ratio of buildings to non-buildings is 3 to 7; (**c**) the ratio of buildings to non-buildings is 4 to 6; (**d**) the ratio of buildings to non-buildings is 5 to 5; (**e**) the ratio of buildings to non-buildings is 6 to 4; and (**f**) the ratio of buildings to non-buildings is 7 to 3.

The results show a striking effort of the ratio of building pixels to non-building pixels on performance in test. From Figure 6, it can be intuitively seen that when the ratio of building pixels to non-building pixels is 4 to 6, the result of the experiment is the best. Less than this ratio, the building pixels are not extracted, and larger than the ratio is a large number of non-building pixels are divided into building pixels. This is because the encoder network fully learns the characteristics of the building area and non-building areas. Consistent results can also be obtained from in Table 1.

### 3.3. Impact of the Different Size of Training Samples

**Figure 7:**
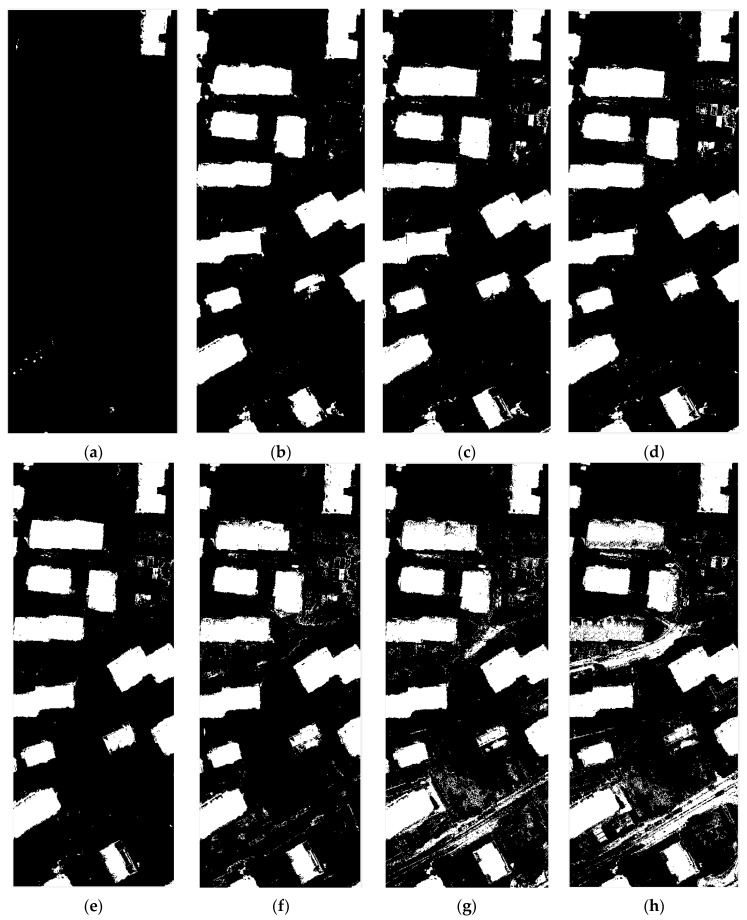
Results of different size of extraction: (**a**) 1000 points; (**b**) 6000 points; (**c**) 10,000 points; (**d**) 15,000 points; (**e**) 30,000 points; (**f**) 100,000 points; (**g**) 150,000 points; and (**h**) 600,000 points.

**Figure 8:**
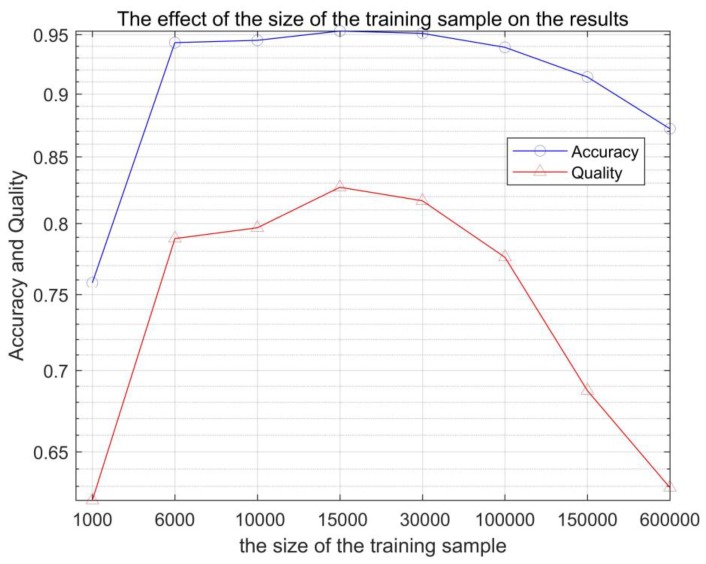
The effect of the size of the training sample on the results.

The ratio of building and non-building training samples was randomly selected according to the proportion of 4 to 6 constituting the training data, and the size of the training data was changed to discuss the effect of the training data on the experimental results. The experimental data were selected in different cases (from 1000, 6000, 10,000, 150,000, 30,000, 100,000, 150,000, and 300,000 training sample points). As shown in Figure 7 and Table 2, the size of the training sample has a certain effect on the detection. From 1000 training sample points and 300,000 training sample points in Figure 8, it can be seen that excessive training samples can cause some non-building pixels to be divided into building pixels, and training samples that are too small can cause the building pixels to be divided into non-building pixels. A suitable training sample can obtain the best results.

### 3.4. Impact of the Different Network Structure

In order to get a more obvious result, the use of a group of poor results of the data, the experiment was carried out under adverse conditions.

**Figure 9:**
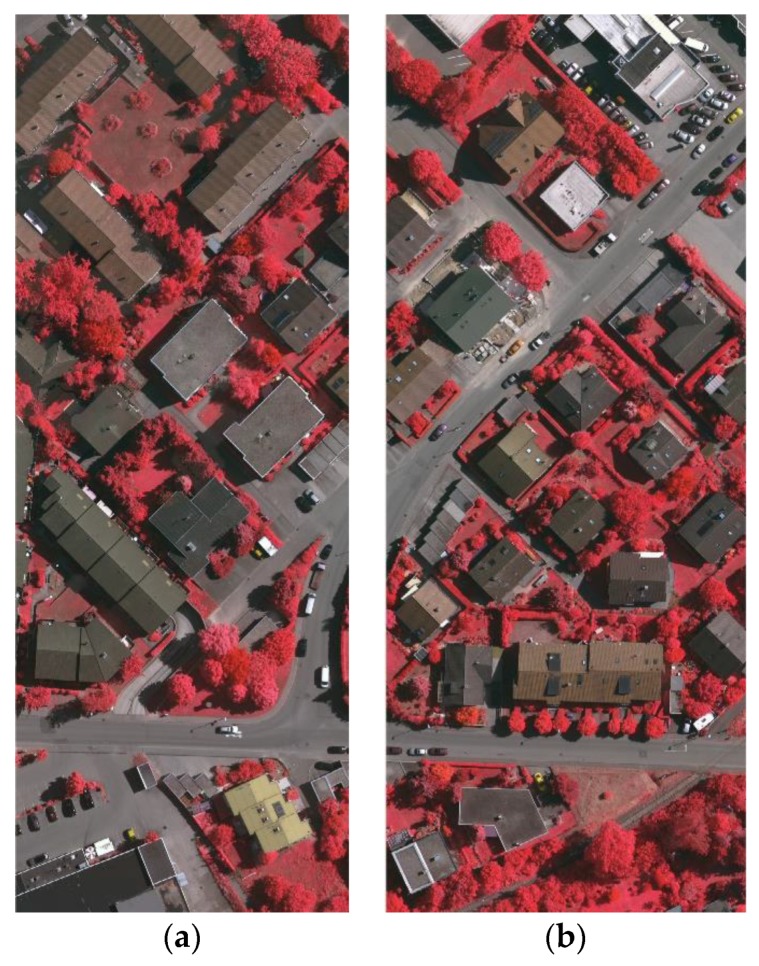
The area of the experiment data is selected: (**a**) the training data is selected on the left side of Area 30; and (**b**) the test data is selected on the right side of zone 30.

As shown in the Figure 9, the entire Area 30 is divided into two parts, the left part of the steps are in accordance with the above steps to build the training samples: that is, according to the ratio 4 to 630,000 points are selected, and the right part of the whole map as a test sample.

Table 3 shows the comparison about the different number of layers of the neural network. When the numapoch and batchsize are the same, the greater number of hidden layers of the neural network show better results.

Table 4 shows the effect of the trend of neuronal changes in the hidden layer on the results when the number of hidden layers is the same. When the number of neurons in the hidden layer decreases by layer, the result is the better. When the number of neurons in the hidden layer is constant, and the number of neurons in the hidden layer is increasing, the effect is not good.

### 3.5. Comparison of Different Methods

We compare stacked sparse autoencoder with a deep Belief Network (DBN) and Support Vector Machine (SVM) to verify that the selected samples and network structures are appropriate. To attain these aims, the most appropriate training data were used in Area 30, Area 34 and Area 37, respectively, and the whole graph was tested for comparison.

SVM parameters were set to *c* = 3.0314; *g* = 84.4485. DBN chose two hidden layers of the network, the first and second hidden layers have 200 and 100 hidden units, respectively. In more than two hidden layers of the moment, DBN will be a fitting phenomenon. The stacked sparse autoencoder network structure contains five hidden layers, the hidden layers have 160, 100, 40, 20 and 10 hidden units, respectively.

Using the same settings of data, training data and test data from the same image, the size of the training data is 30,000, the size of test data is 2,500,000. In the training data, the ratio between the building and the non-building is 4 to 6.

**Figure 10:**
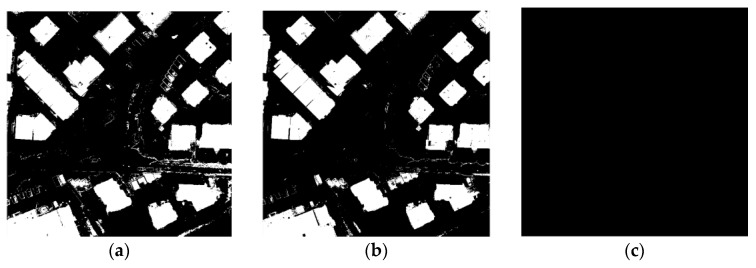
The result of Area30: (**a**) The result of DBN; (**b**) The result of SSAE; (**c**) The result of SVM.

**Figure 11:**
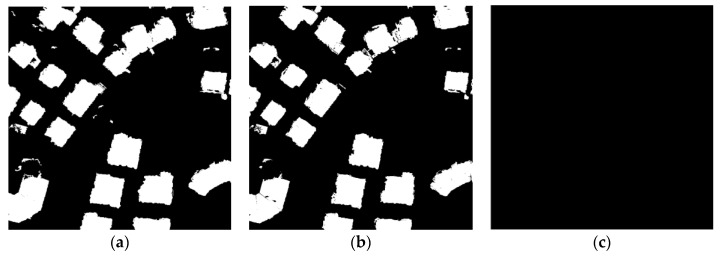
The result of Area37: (**a**) the result of DBN; (**b**) the result of SSAE; and (**c**) the result of SVM.

**Figure 12:**
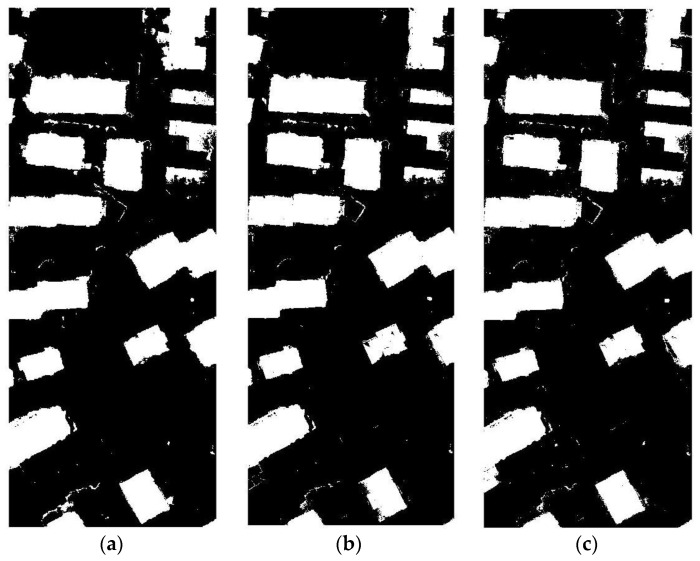
The result of Area 34: (**a**) the result of DBN; (**b**) the result of SSAE; and (**c**) the result of SVM.

The results of Area 30 and Area 37 were shown in Table 5 and Table 6 and Figure 10 and Figure 11, it could be found that with above settings, SSAE and DBN could get a good accuracy, but SVM will be the whole image to determine the non-buildings though parameters optimization were done. The reason could be over-fitting. SVM in the treatment of such data will appear such an unstable situation. Table 7 and Figure 12 show the comparison of the DBN, SSAE, and SVM in Area 34, respectively. We can see that the results of SVM is the best, but it takes almost five times the time of SSAE. The DBN method works the worst. SSAE results better than DBN, and spend almost the same time.

## 4. Discussion and Conclusions

In this paper, we proposed a remote sensing building extraction method, based on an effective stacked sparse autoencoder network, which combines the original optical and DSM data as input and extract the buildings in the high-resolution data. Although there are many feature-based or fusion methods might obtain better performance than SSAE, we believe SSAE can find more abstract and unusual features. Moreover, finding an optimized using idea of SSAE is far-reaching significance. As a result, we mainly focused on discussing two problem on remote sensing building extraction: (1) the suitable size and proportions of training samples for deep learning based methods; (2) the better structure of SSAE based methods. According to our experiments, we found appropriate settings of the training sample size was 15,000 points, and the better proportion between the building and the non-building was 4:6. For the structure of SSAE, according to our experiments, the number of neurons in the hidden layer should be decreased set layer by layer, and the most suitable number of layers is 5, with 160-100-40-20-10 hidden units, respectively.

In our idea, the units composing different structure of SSAE are not only highly isolated abstract features, but can also be considered as a method of thinking to do one specific thing, the layers can be the levels of thinking, and the values of each unit is the weight of one of some unsure factor. In the experiments, better results were obtained by the decreased set structure, so it could be deduced that sub-total way of thinking is suitable for building detection with LIDAR DSM and optical images, and the sub-total way of thinking can be thought as the unusual features of this application. Moreover, five levels of thinking with corresponding weights of each factors is an ideal trade off. Furthermore, it is also important that not each isolated factor itself determine the result directly, but the way of thinking constructed by the SSAE which can lead to an ideal processing for some specific applications, with some appropriate number and proportions of prior samples to train the way of thinking.

## Figures and Tables

**Table 1:** Different proportions of extraction results.

	2:8	3:7	4:6	5:5	6:4	7:3
Accuracy	86.79%	93.01%	93.93%	89.52%	68.33%	67.03%
quality	48.49%	73.40%	77.07%	68.49%	43.23%	42.51%

**Table 2:** The results of different sizes of training samples.

	1000	6000	10,000	15,000	30,000	100,000	300,000	600,000
Accuracy	75.8%	94.3%	94.5%	95.3%	95.1%	93.9%	91.4%	87.2%
quality	6.22%	78.9%	79.7%	81.6%	82.6%	77.5%	68.7%	58.7%

**Table 3:** Different proportions of extraction results.

Network Structure	Numepochs	Batchsize	Accuracy	Quality
[160 100 40 20]	50	100	92.12%	70.0%
[160 100 40]	50	100	90.87%	67.2%
[100 40 20]	50	100	90.40%	66.3%
[160 40 20]	50	100	91.08%	65.6%
[160 100]	50	100	90.39%	65.0%
[160 40]	50	100	84.80%	55.7%
[100 40]	50	100	90.36%	64.7%
[40 20]	50	100	89.31%	62.9%
[160]	50	100	86.93%	57.7%
[100]	50	100	88.04%	59.6%
[40]	50	100	87.68%	58.9%
[20]	50	100	87.99%	60.1%

**Table 4:** Different proportions of extraction results.

Network Structure	Numepochs	Batchsize	Accuracy	Quality
[160 100 40 20]	50	100	92.12%	70.0%
[20 40 100 160]	50	100	89.27%	63.8%
[40 40 40 40]	50	100	88.35%	61.6%
[100 100 100 100]	50	100	88.35%	61.3%
[160 40 20]	50	100	91.08%	65.6%
[20 40 160]	50	100	89.54%	63.6%
[160 100 40]	50	100	89.87%	67.2%
[40 100 160]	50	100	88.62%	62.2%
[160 160 160]	50	100	87.07%	59.1%
[100 100 100]	50	100	89.20%	63.2%
[40 40 40]	50	100	88.15%	61.1%
[20 20 20]	50	100	88.82%	62.1%

**Table 5:** Different methods of extraction results Area 30.

	Accuracy	Completeness	Correctness	Quality	Time
DBN	92.46%	89.49%	83.20%	75.79%	21.30 s
SSAE	94.20%	86.90%	90.73%	79.81%	23.60 s
SVM	73.63%	100%	73.63%	73.63%	3650.33 s

**Table 6:** Different methods of extraction results.

	Error Rate	Completeness	Correctness	Quality	Time
DBN	94.36%	92.56%	86.65%	81.01%	18.73 s
SSAE	95.45%	90.71%	90.98%	83.22%	18.61 s
SVM	75.20%	100%	75.20%	75.20%	4247.86 s

**Table 7:** Different methods of extraction results of Area 34.

	Accuracy	Completeness	Correctness	Quality	Time
SSAE	96.23%	94.71%	91.07%	86.66%	21.47 s
DBN	94.28%	85.38%	92.37%	79.76%	22.21 s
SVM	97.49%	98.18%	98.43%	96.66%	110.21 s
