# Correction: Yan, Y.; et al. Building Extraction Based on an Optimized Stacked Sparse Autoencoder of Structure and Training Samples Using LIDAR DSM and Optical Images. *Sensors* 2017, *17*, 1957

**DOI:** 10.3390/s19010131

**Published:** 2019-01-02

**Authors:** Yiming Yan, Zhichao Tan, Nan Su, Chunhui Zhao

**Affiliations:** College of Information and Communication Engineering, Harbin Engineering University, Harbin 150001, China; zhaochunhui@hrbeu.edu.cn

The authors wish to make the following corrections to this paper [1]:

Changes in Acknowledgement

Due to a lapse, some important contents were missed in the Acknowledgement section from the original article [1].

[**Acknowledgments**: The authors would like to thank the support by the Fund of the National Natural Science Foundation of China under Grant No. 61601135 and Natural Science Foundation of Heilongjiang Province of China under Grant No. QC201706802. Additionally, the Vaihingen data set was provided by the German Society for Photogrammetry, Remote Sensing and Geoinformation (DGPF) [Cramer, 2010]: http://www.ifp.uni-stuttgart.de/dgpf/DKEPAllg.html. The experimental SVM program comes from libsvm-3.1-[FarutoUltimate3.1Mcode]. Faruto and liyang, LIBSVM-faruto Ultimate Version, a toolbox with implements for support vector machines were based on libsvm, 2011. Software is available at http://www.matlabsky.com. Chih-Chung Chang and Chih-Jen Lin, LIBSVM: a library for support vector machines, 2001. Software is available at http://www.csie.ntu.edu.tw/~cjlin/libsvm. The experimental SAE program comes from the rasmusbergpalm-DeepLearnToolbox-9faf641 toolkit. Software is available at https://github.com/rasmusbergpalm/DeepLearnToolbox.]

The authors would like to apologize for any inconvenience caused to the readers by these changes.
